# Actor-Network Theory: Insights into the Study of Social-Ecological Resilience

**DOI:** 10.3390/ijerph192416704

**Published:** 2022-12-13

**Authors:** Song Yao, Kui Liu

**Affiliations:** School of Marxism, Southeast University, Nanjing 210037, China

**Keywords:** actor-network theory, social-ecological resilience, Chang-shan Archipelago, thematic analysis, adaptive governance

## Abstract

Actor-network theory, which emerged from science and technology studies in the 1980s, regards everything in the social and ecological systems as a continuous result of the network of relations where they are located. Social-ecological resilience, with its origins in systems ecology, focuses on the non-linear changing dynamics of social-ecological systems and their governance. Among them, social-ecological resilience study integrates different disciplines, backgrounds, and themes, which inevitably leads to the vagueness of its concept. Both actor-network theory and social-ecological resilience emphasize human-nature relationships and view social-ecological systems as dynamic and unpredictable “networks”. Therefore, this paper explored the potential conceptual or theoretical underpinnings that actor-network theory can provide in social-ecological resilience through interdisciplinary research. Specifically, a semi-structured interview was conducted with 30 fishing households from Chang-shan Archipelago in Northeastern China. The obtained interview data were analyzed through thematic analysis, and three main themes were generated, including “heterogeneous networks”, “agency”, and “translation”, which facilitated a reconceptualization of the three components of social-ecological resilience, namely, “linked social-ecological systems”, “changing dynamics” and “the ability to maintain resilience”, and also provided a new theoretical perspective on the adaptive governance of social-ecological systems.

## 1. Introduction

A growing number of scholars and policymakers believe that the interaction and interdependence of social and ecological systems provide a promising tool for guiding actions toward a more sustainable society [1]. Therefore, the study of social-ecological resilience has obtained more and more attention. However, the integration of different disciplines, backgrounds, and themes inevitably leads to conceptual vagueness in social-ecological resilience [2]. At the same time, the social dimension is often overlooked in social-ecological resilience studies, which weakens the analysis of interactions between social and ecological systems. But it is noteworthy that some unique dimensions of the social system, such as historic, cultural, and political contingencies, as well as human agency and normative issues, can deepen the understanding of social-ecological resilience [3,4]. Therefore, it is necessary to reconceptualize social-ecological resilience by using social sciences to make its concept more comprehensive and explicit.

Over the past decade, significant achievements have been made in the interdisciplinary integration of social sciences and social-ecological resilience. For example, Hobman et al. introduced psychological research methods into the study of social-ecological resilience. By combining Levin’s field theory with the latest contributions of ecology, cognitive science and social psychology, they developed a conceptual framework to further understand social-ecological resilience through the intersection between subjective psychology and objective environment [5]. Fabinyi et al. discussed the importance of introducing power and social diversity into social-ecological resilience from the perspective of ecological and social anthropology and political ecology. They proposed that a better understanding of power relations in social-ecological systems is conducive to deconstructing whose voices are privileged, whose voices are marginalized, and why [6]. Therefore, it is important to go beyond the perspective of current social units and institutions to understand the inherent complexity and diversity between ecology and society. Later, Boonstra further proposed the concept of social power on this basis, which is beneficial to comprehend the impact of power on social-ecological interaction [7]. He cited the domestication of fire as an example to illustrate how hominids used fire as a source of social power to reconstruct the relationship between human beings and the environment. The above studies introduced social sciences into social-ecological resilience by strengthening the debate and inquiry between the two, which deepens the understanding of the complex interaction between social and ecological systems, and provides a new perspective and approach to studying social-ecological resilience.

As an important social science, actor-network theory offers provocative insights into the interdisciplinary integration of social sciences and social-ecological resilience [4]. Firstly, the actor-network theory provides a diverse perspective for social-ecological resilience study, not only considering the role of human and non-human actors in social-ecological phenomena and changes but also transcending the binary thinking such as local and global, subject and object, which provides a unique and valuable framework for solving practical problems such as how human and non-human actors maintain social-ecological resilience. Secondly, the integration between actor-network theory and social-ecological resilience is well-documented. Both actor-network theory and social-ecological resilience focus on human-nature relationships and regard the social-ecological systems as dynamic, changing, and unpredictable “networks” [8,9]. Thirdly, given that social-ecological resilience study has not formed a solid theoretical basis for how different actors maintain resilience, this is particularly important. Therefore, the actor-network theory is still instructive for studying social-ecological resilience.

However, previous interdisciplinary research between social sciences and social-ecological resilience still exhibits fragmentation and superficiality [1,10], emphasizing only theoretical analysis but ignoring actual case analysis, which weakens their persuasiveness. Therefore, it is necessary to further explore the interfaces between the two and achieve better integration by combining specific case analyses, thus making a contribution to the study of social-ecological resilience. Therefore, this paper first gave a comprehensive overview of actor-network theory and social-ecological resilience. Secondly, a semi-structured interview was conducted with 30 fishing households, and the interview data were analyzed through thematic analysis to explore the potential conceptual or theoretical underpinnings provided by actor-network theory in reconceptualizing social-ecological resilience. Thirdly, a new theoretical perspective offered by actor-network theory in the adaptive governance of social-ecological systems was discussed.

## 2. An Overview of Social-Ecological Resilience and Actor-Network Theory

### 2.1. An Overview of Social-Ecological Resilience

Social-ecological resilience originates from systems ecology, focusing on the non-linear changing dynamics of social-ecological systems and their governance. Holling first introduced the concept of “resilience” into ecology research and defined it as “a measure of the ability of these systems to absorb changes of state variables, driving variables, and parameters, and still persist [11]”. In the following decades, resilience research gradually extended from natural ecology to human ecology and other fields such as cities, economies, and societies. Berkes et al. used the social-ecological system as a comprehensive perspective to analyze human-nature relationships and connected it with the emerging concept of “resilience” at that time [12]. Carpenter et al. formally proposed social-ecological resilience based on the interaction between human beings and ecological systems [13]. Social-ecological resilience is defined as “the capacity of a social-ecological system to absorb disturbance and reorganize while changing to retain essentially still the same function, structure, and feedback” [14,15]. Among them, the absorption of disturbance refers to the amount of change that a system can absorb without changing its state, and reorganization refers to the ability of a system to respond to changes through the interaction of its components [16]. If a system can absorb disturbance and reorganize, then it is resilient. The three components of social-ecological resilience are “linked social-ecological systems”, “changing dynamics”, and “the ability to maintain resilience”. According to the definition of social-ecological resilience, the relationship between social and ecological systems is interrelated and continuously interactive, interacting at multiple temporal and spatial scales [17]. The changing dynamics of the system conform to the four phases of exploitation, conservation, release, and reorganization specified in the adaptive cycle [18]. The transition between each phase depends on three dimensions: potential, connectedness, and resilience [19]. In the exploitation phase, the resources and relationships of the system experience a faster accumulation, and resilience gradually increases, while potential and connectedness do not change significantly. As the system enters the conservation phase, the rate of accumulation slows down, and the system reaches a quasi-equilibrium condition. Meanwhile, the system’s potential, connectedness, and resilience continue to increase and achieve high levels. In the release phase, the system quickly releases the accumulated resources, the potential and resilience of the system decrease rapidly, and connectedness also decreases to a certain degree. In the reorganization phase, the system’s potential reaches a high level. Through continuous learning, capital accumulation and adaptive capacity, the resilience and connectedness of the system gradually increase [20].

Since social-ecological resilience was proposed, there have been two shifts in its research focus. At first, social-ecological resilience was mainly explored at a regional level. For example, Berkes et al. discussed the social-ecological resilience in a Canadian Western Arctic Community during the process of adapting to climate change, which originated from the ability of local people to deal with a changing and uncertain environment over a long time [21]. Later, scholars began to explore social-ecological resilience at a global level. Rockström et al. proposed a framework based on “planetary boundaries”, which defined a safe operating space for human beings with respect to the Earth system. The complex system of the Earth can respond to changing external shocks within the threshold and remain resilient, but once the threshold is crossed, it may bring about disastrous consequences for human beings [22]. Adger et al. analyzed the impact of global marine disasters on social-ecological resilience. Considering the trend of human communities, resource utilization, and global environmental change, it is urgent to build resilience in coastal areas, which requires a broader understanding and cultivation of social-ecological resilience [23]. In recent years, social sciences have been gradually introduced into the study of social-ecological resilience. For example, Stone Jovicich et al. introduced social sciences such as material-spatial world systems analysis and critical realist political ecology into social-ecological resilience, which enhanced the explanatory power of social-ecological resilience to social and environmental changes [4]. Hobman et al. applied Levin’s field theory to the study of social-ecological resilience, which contributed to the understanding of coupled social-ecological systems. The field theory captures the stasis and change of the system from both external drivers and internal forces, which coincides with some propositions of social-ecological resilience [5].

### 2.2. An Overview of Actor-Network Theory

Actor-network theory is a social theory proposed by Bruno Latour, Michel Callon, and John Law in the mid-1980s [24], its intellectual and historical roots stem from the Innovation Sociology Center of the French College of Mining and have been influenced by the emergence of the French post-structuralism and the debate on the sociology of science [25]. Actor-network theory tried to replace the traditional binary thinking with relational ontology and break the boundaries between nature and society [26], regarding all the entities constantly circulating, connecting, and reshaping their identities in the network [27]. The three core concepts of actor-network theory are actor, network, and translation, which are initially used to explain the process of knowledge production. Firstly, actors are heterogeneous, including human beings and non-human actors (such as natural objects, artificial objects, ideas, and concepts), and both have agency simultaneously. Secondly, a network refers to a dynamic and inseparable heterogeneous actor network constructed by the interactive practice between human and non-human actors. Among them, non-human actors are no longer merely material resources or constraints but are actively involved in the action. The connections between heterogeneous actors make them have relational materiality, which endows them with the qualification to participate in network construction [24,28]. Thirdly, heterogeneous actors are connected through translation. The translation establishes connections, links different actors, and creates convergences by forming a channel between other domains [29]. Specifically, the key actor continues to translate the interests and problems of other actors into his language. Through translation, actors can be connected and form a network.

Since the 1980s, the focus on actor-network theory has shifted three times. Initially, the actor-network theory was applied to the micro-level research of “science in action”. Since the beginning of the 1990s, the focus of actor-network theory research has gradually shifted to the field of technology. The main concepts of actor-network theory can provide new theoretical and methodological underpinnings for understanding the production process of technology [30]. Taking information technology as an example, its birth went through complex technological and social processes, which were translated and written into specific entities and formed inscriptions. The irreversibility of inscription makes its network stable in different contexts. Therefore, technology has the dual attributes of “individual” and “collective”, which makes up for the binary opposition between technology and society [31]. Since the late 1990s, globalization has become a new research focus for actor-network theory. Smith et al. put forward world city actor-networks and analyzed the relationship between world cities from the perspective of actor-networks [32]. As nodes of the network, cities communicate and interact with each other, which effectively promotes the process of globalization and overcomes the binary opposition between local and global. In recent years, scholars have gradually diverted to “ontology” and “materiality” research [31]. Actor-network theory adheres to relational ontology, which insists that human and non-human actors’ identities and attributes are determined by their interconnections in the network [33,34]. Therefore, actors exist in the form of “other”, which can be distinguished from the previous view that actors exist in the form of “being” [35]. In addition, according to actor-network theory, materials are generated in a specific network of relations and are inevitably intertwined with others. This new definition of material can resonate with new materialism and transcend traditional materialism [36]. However, since the actor-network theory was proposed, it has gained significant popularity but has also encountered some doubts [37,38]: firstly, how to define human and non-human actors; secondly, whether non-human actors have agency and how to exert their agency; and thirdly, how to translate the interests and problems of non-human actors. Finally, whether the equal treatment of all actors ignores the importance of specific actors. Nevertheless, this cannot deny the theoretical innovation and value that actor-network theory has brought to the development of social sciences.

## 3. Methods

### 3.1. Study Design

**Figure 1:**
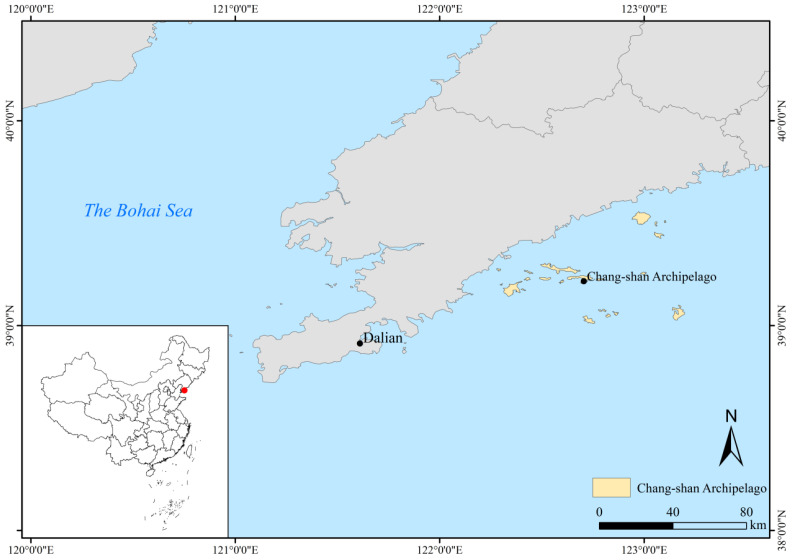
Location map of Chang-shan Archipelago.

This study aimed to explore the potential theoretical underpinnings that actor-network theory can provide in reconceptualizing social-ecological resilience. On this basis, 30 fishing households from the Chang-shan Archipelago (see Table 1 for details) were invited to conduct a semi-structured interview, and the interview data were analyzed by thematic analysis. Among them, Chang-shan Archipegalo is located in the Northern part of the Yellow Sea and the Southern part of the Liaodong Peninsula and consists of islands such as Shicheng Island, Dachangshan Island, Xiaochangshan Island and Guanglu Island et al., which is 1 of the 8 major archipelagos in China (see Figure 1). The leading industries of Chang-shan Archipelago mainly include mariculture, fishing, aquatic products processing and island tourism.

### 3.2. Data Collection

The interview was conducted in the homes of 30 fishing households in the Chang-shan Archipelago. Based on the research questions, a semi-structured interview outline was developed, mainly including introductory, flow, key and final questions (see Table 2 for details) [39]. The introductory questions were designed to make interviewees adapt to the atmosphere and rhythm of the interview more quickly. The flow questions were designed to create a smooth transition to the key questions the researchers wanted to explore. The final questions were designed to summarize the interviews and ensure that interviewees had no additional comments.

### 3.3. Data Analysis

Firstly, the interview data were collated. Secondly, the open coding of interview data was conducted through thematic analysis (see Table 3 for details) [40,41]. The codes screened from the interview data can be used as a centralizing organizing concept of theme. A theme was defined as something that has a certain level of pattern or meaning in relation to the research questions in the data [42].

## 4. Findings

Three themes and 10 sub-themes were identified in this study, as shown in Table 4. The three themes are “heterogeneous networks”, “agency”, and “translation”, which provide some inspiration for reconceptualizing the three components of social-ecological resilience, namely, “linked social-ecological systems”, “changing dynamics”, and “the ability to maintain resilience”.

### 4.1. Insights of “Heterogeneous Networks” into Reconceptualizing “Linked Social-Ecological Systems”

In 1998, Berkes and Folke first proposed the social-ecological systems framework [12]. They believed that social and ecological systems are actually related, and the separation between the two is artificial and deliberate, but they did not define linked social-ecological systems. In the next 20 years, scholars such as Andries [43], Ostrom [44,45], and Fidel [46] tried to define linked social-ecological systems. Among them, Andries et al. [43] made a relatively more comprehensive and explicit definition of linked social-ecological systems, namely, “an ecological system intricately linked with and affected by one or more social systems”, but this definition has not overcome the binary opposition between ecology and society. At present, confronted with the vagueness of the concept of linked social-ecological systems, it is necessary to conduct more in-depth interdisciplinary research. Actor-network theory can reconceptualize “linked social-ecological systems” from the perspective of “heterogeneous networks”.

Actor-network theory tried to break the conventional barriers between social and ecological systems and transcend the traditional dichotomy of nature and society by establishing actor networks to rebuild the internal connection between binary factors. According to actor-network theory, all actors in the social and ecological systems are aligned in networks of different scales and form inextricably linked heterogeneous networks of human and non-human actors. As a node, all actors have inter-subjectivity and are equal to each other. They coordinate and “weave” an actor network without center or subject-object dichotomy. Among them, what matters is the process of networking (that is, how human and non-human actors gather together and interact with each other) and how the actors are affected when they interact with each other [47]. Therefore, social and ecological systems can be regarded as heterogeneous networks that are interdependent and continuously interacting. Then ecology is branded with the characteristics of society, and society is also branded with the characteristics of ecology. As a result, social and ecological systems cannot be artificially separated. Latour’s “Parliament of Things” vividly depicts heterogeneous networks. In a “Parliament of things”, all actors are given the legitimacy to participate in the “Politics of nature”, and they can all be adequately represented and speak in their own names [48].

The social-ecological system of the Chang-shan Archipelago can also be seen as an interrelated and interdependent heterogeneous network composed of different actors. In the process of mariculture and fishing, fishermen, government officers, technical professionals, marine creatures and fishing technologies formed an inseparable network. As can be seen from the interview, when asked about the contact with other fishermen, government officers, technical professionals and marine creatures in the process of mariculture and fishing, 28 interviewed fishing households said they had close contact with them. For example, “Fishermen rely on the sea to live, and when there is more fish in the sea, there is more harvest”. “Usually, we go fishing with other fishermen, and the floating rafts are often purchased and used together by two fishing households”. “The local government officers regularly go to Dalian to publicize and expand the sales channels of oysters”. “Technical professionals from Dalian Ocean University regularly come here to transfer mariculture knowledge and develop some solutions to specific problems”. From the above analysis, it can be found that in the social-ecological system of Chang-shan Archipelago, a “heterogeneous network” was formed between fishermen, government officers, technical professionals, marine creatures, and fishing technologies. The interdependence between different actors has effectively improved the ability of fishermen to cope with extreme weather and their living standards.

### 4.2. Insights of “Agency” into Reconceptualizing “Changing Dynamics”

Over time, the structures and functions of social-ecological systems change due to internal dynamics and external influences [49]. Holling described the changing dynamics of social-ecological systems as four sequential phases: exploitation, conservation, release, and reorganization [50,51], which can be seen as an adaptive cycle. However, the adaptive cycle metaphor does not explain the changing dynamics of all systems. For example, it cannot be applied to explain a release phase involving no loss of capital. During the transition from a bog to a forest, the ability of the bog to self-organize and resist invasion by trees is eventually overwhelmed, and the reorganization into a forest occurs, but without any creative destruction phase involving a loss of nutrients [49]. Based on the adaptive cycle, resilience scholars have developed an alternative theory named panarchy to capture and explain the changing dynamics of social-ecological systems. The panarchy represents the dynamic interplay between processes and structures that sustain relationships on the one hand and create and accumulate potential on the other [52]. Although panarchy can be used to analyze and explain the changing dynamics of social-ecological systems, some scholars have expressed concerns about it. For example, Chaffin et al. proposed that the combination of panarchy theory and social sciences is more conducive to clarifying the vulnerability and resilience dynamics in social-ecological systems [52]. Actor-network theory can reconceptualize “changing dynamics” from the perspective of “agency”.

According to actor-network theory, both human and non-human actors have agency, which qualifies them to play a role in the social-ecological system, and inevitably affects the changing dynamics of the whole system. Specifically, human agency is mainly shown as initiative, that is, the ability and willingness to actively take action. Non-human actors cannot take the initiative but can have an impact on the system’s functioning. This paper attempts to analyze the influence of human and non-human actors’ agency on the changing dynamics of the system by defining the changing dynamics of the social-ecological system of the Chang-shan Archipelago, especially in some special situations that cannot be explained by the adaptive cycle.

**Figure 2:**
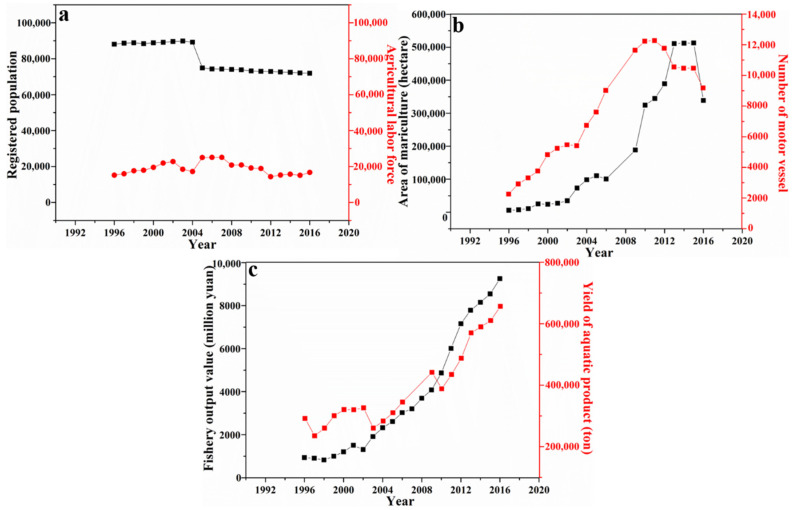
Year-to-year changes in (**a**) registered population and agricultural labor force; (**b**) area of mariculture and number of motor vessels; (**c**) fishery output value and yield of aquatic product.

In order to analyze the changing dynamics of a social-ecological system, some key variables of social and ecological dimensions need to be selected. According to the research of Gunderson and Holling [53], this paper selected the population, fishery output, and marine resource utilization from 1996 to 2016 as key variables to analyze the changing dynamics of the social-ecological system in the Chang-shan Archipelago. Firstly, in terms of population, the registered population showed a significant downward trend from 88,069 to 71,928. Among them, the population engaged in agriculture (mainly fishing) increased slightly from 15,164 to 16,708, remaining roughly stable, as shown in Figure 2a. In terms of marine resource utilization, the mariculture area increased from 86,842 to 338,679 hectares, and the number of motor vessels increased from 2231 to 9160, as shown in Figure 2b. In terms of fishery output, the yield of aquatic products increased from 291,820 to 656,614 tonnes, and the fishery output value increased from 937 million yuan to 9255 million yuan, as shown in Figure 2c. To sum up, from 1996 to 2016, the population engaged in agriculture (mainly fishing) remained roughly stable, and the mariculture area and the number of motor vessels increased significantly. As a result, the development potential of fishing has improved significantly, and the fishery output value has also experienced significant growth. Therefore, in the past two decades, the potential, connectivity, and resilience of the social-ecological system of the Chang-shan Archipelago have increased. The system has experienced the transition from the exploitation phase to the conservation phase, which conformed to the adaptive cycle metaphor.

As components of a social-ecological system, both human and non-human actors have the agency to speed up or slow down the accumulation of the system’s potential, connectedness and resilience, thus shortening or lengthening the time needed to shift from one phase to another, or even leading to special situations that do not fit the characteristics of the current phase. For example, since 2011, the number of motor vessels has decreased year by year. Since 2012, the mariculture area remained stable for 3 years and then decreased rapidly. The reason for the above phenomenon is that, since 2011, the local government has taken a number of environmental protection measures to restore the local ecological environment in response to increasingly serious marine pollution and fishery resource reduction. On the one hand, the local government began to restrict excessive fishing and strictly control the issuance of motor vessel licenses. On the other hand, the government began to promote intensive aquaculture and reduce the mariculture area. However, from 2012 to 2016, the yield of aquatic products and fishery output value of Chang-shan Archipelago still experienced relatively rapid growth. The reasons for the above phenomenon are that, on the one hand, the number of marine creatures has significantly increased through government-organized release activities and fishing moratoriums. On the other hand, the improvement of oyster and scallop varieties and mariculture technology have improved their survival rates and production. To conclude, with the implementation of environmental policies, the regulation of unreasonable fishing modes and the application of advanced technologies, the accumulation of the system’s potential and resilience has been accelerated. As seen from the interview, 21 interviewed fishing households said that the government’s fishing moratorium had effectively improved the marine environment and increased the fishing yield. For example, “After the fishing moratorium, it is obvious that the seawater has become clear, and the fish are bigger”. Nineteen interviewed fishing households said that after the release activities organized by the government, they caught more kinds of fish. For example, “I caught two bastard halibuts this year that had not been seen for decades”. Twenty-seven interviewed fishing households stated that improvements in fishing technologies had significantly increased fishing output. For example, “The improvement of seedling technology has significantly improved the survival rate of sea cucumbers”. “Since we started to breed the third-generation oysters, the quality and yield have improved significantly, and the market has also expanded”. Therefore, both human actors (such as government officers) and non-human actors (such as fishing technologies and marine creatures) are important factors that affect the changing dynamics of a system. Therefore, it is necessary to consider the actors’ role when analyzing a system’s changing dynamics, especially in some special situations.

### 4.3. Insights of “Translation” into Reconceptualizing “the Ability to Maintain Resilience”

The ability of a system to self-organize, learn and adapt are two critical indicators for maintaining social-ecological resilience, which can make the social-ecological systems absorb external disturbance and keep in a stable zone [54]. The ability to self-organize refers to the power of a system to organize itself when encountering external disturbances, and the ability to learn and adapt refers to the power of system members to adapt to changes and maintain resilience by acquiring knowledge and skills [55]. It has been proved that the ability to self-organize, learn and adapt is conducive to a social-ecological system to cope with challenges and maintain resilience. Improving the ability to self-organize, learn and adapt among actors can make social-ecological systems cope with the risk of food shortage and extreme weather events more effectively [56,57,58]. In addition, it can also reduce the vulnerability of social-ecological systems and increase resilience. For example, the experience in dealing with natural disasters has provided a window of opportunity for new policy formulation on disaster prevention and mitigation, which facilitates the prediction of the frequency and extent of disasters and the reduction of their impact [59,60]. Some scholars have analyzed the importance of biodiversity in maintaining the resilience of social-ecological systems. Biodiversity provides cross-scale resilience, bringing different species together to form a set of overlapping and reinforcing effects, which can spread risk, enhance ecological function and reduce vulnerability to large fluctuations in specific species [61,62]. Di Falco et al. demonstrated that increased biodiversity could improve the ecological system’s tolerance and reduce the negative impacts of natural disasters such as rainfall shocks [63]. Actor-network theory can reconceptualize “the ability to maintain resilience” from the perspective of “translation”.

According to actor-network theory, human and non-human actors form a network through translation. A smooth translation can effectively increase the network’s resilience, enabling it to absorb external disturbance and keep it in a stable zone. Firstly, all actors have common interests, which is the premise of building a resilient network. Specifically, the key actor translates the desires and demands of other actors, realizes the association of different interests, and forms a resilient network. Secondly, actors have the ability to maintain resilience. Human actors play a leading role in maintaining social-ecological resilience, determining the desired configurations of social-ecological systems [64], shaping governance systems [65], and enhancing resilience to environmental change [66,67,68]. Non-human actors cannot consciously participate in the maintenance of social-ecological resilience. As members of social-ecological systems, non-human actors participate in the system construction process as mediators, which benefits the maintenance of social-ecological resilience [69,70]. Thirdly, the network enters a stable state when translation is completed, and symmetry is achieved. An effective and smooth translation process can attract more actors to participate in network construction, which contributes to the interaction between actors and the increase of resilience [71,72]. From the interview, it can be found that 30 interviewed fishing households said that they joined the group of villagers, so they could timely learn about the new policies and express some personal views on the development of the village. For example, “the group of villagers holds regular meetings, and the villagers’ representatives collect the fishermen’s opinions and discuss them at the meeting”. “The new policies issued by the government will be timely conveyed at the villagers’ meeting”. At the same time, 30 interviewed fishing households said they joined the WeChat group of fishermen, where they can often get some mariculture experience and weather warning information. For example, “We would share some new scallop and oyster varieties and mariculture experience in the WeChat group of fishermen”. “The WeChat group of fishermen would share a gale warning a few days in advance so that we can make preparations to reduce some losses”. Sixteen interviewed fishing households said they participated in fishery cooperatives, so that they can buy fishing machines, sea cucumber and oyster seedlings through collective bargaining to obtain preferential prices. For example, “After joining the fishery cooperative, we can buy sea cucumber seedlings at a more favorable price, and the scale of mariculture has also expanded”. In conclusion, the connections between fishermen, government officers and technical professionals have become closer, and the translation between different actors has become more convenient and smooth through joining the group of villagers, WeChat group of fishermen and fishery cooperatives, which effectively improves the resilience of the entire social-ecological system in the face of external disturbances.

## 5. Application of Actor-Network Theory in the Adaptive Governance of Social-Ecological Systems

One of the main dilemmas of the current research on social-ecological resilience is implementing effective governance in a dynamic and non-linear changing relational world. Those centralized systems of governance that rely on top-down instructions can hardly match the scale of ecological complexity, especially in the face of rapid environmental change [73,74]. Therefore, centralized governance systems cannot provide effective solutions for highly contextualized scenarios, their previous performances in the collaborative governance of large-scale ecosystems across multiple jurisdictional boundaries were generally poor [75,76]. As a response to the dilemmas confronted by centralized governance systems, adaptive governance was proposed. Adaptive governance emphasizes inclusion and advocates the participation of multiple subjects in governance, which can flexibly respond to highly contextualized social-ecological systems and adapt to complex and unpredictable feedback among their components. Although the research on adaptive governance has thrived in recent years, it still faces some dilemmas, which are mainly manifested in insufficient combination with other theories and a need for more specific case assistance. The actor-network theory contains the wisdom of adaptive governance and regards governance as a form of social coordination. Specifically, human and non-human actors are integrated into the governance network of social-ecological systems to achieve a pluralistic co-governance. Therefore, no matter whether in the center or on the edges, all actors constitute big or small nodes in the network and can participate in the governance process and make a difference. In the governance of the social-ecological system in Chang-shan Archipelago, different actors (government officers, fishermen, fishing technologies, and technical professionals, et al.) played their respective roles to different degrees, realizing a kind of multi-governance. Firstly, the local government played a leading role in governance. After consultation with the public, expert assessments and democratic deliberations, the local government formulated environmental protection policies, set up a fishing moratorium and organized release activities, which have promoted the recovery and development of the social-ecological system. Secondly, as the subjects of governance, fishermen could timely learn about weather warning information, share their experience in mariculture and fishing, and obtain preferential prices for fishing machines through collective bargaining by joining the group of villagers, WeChat group of fishermen and fishery cooperatives. Thirdly, fishing technologies have improved the quality and yield of aquatic products and thus increased fishermen’s income. Moreover, the promotion of new pollution-free mariculture technology has effectively controlled the trend of marine pollution. Finally, technical professionals would regularly transfer mariculture knowledge to fishermen, effectively solving practical problems in mariculture and fishing. In addition, the introduction of new mariculture varieties and technologies and the expansion of mariculture scales need to be evaluated and reviewed by technical professionals.

## 6. Conclusions

This paper explored the potential theoretical underpinnings of actor-network theory in reconceptualizing social-ecological resilience through a semi-structured interview with 30 fishing households from Chang-shan Archipelago in Northeastern China. A thematic analysis of the interview data was conducted, and three main themes were generated, including “heterogeneous networks”, “agency”, and “translation”, which facilitated a reconceptualization of the three components of social-ecological resilience, namely, “linked social-ecological systems”, “changing dynamics” and “the ability to maintain resilience”, and also provided a new theoretical perspective on the adaptive governance of social-ecological systems. Firstly, the actor-network theory confirmed that social and ecological systems are inseparable networks composed of different actors from the perspective of “heterogeneous networks”. Secondly, the actor-network theory compensated for the deficiencies of the adaptive cycle in explaining some special situations in the changing dynamics of the system from the perspective of “agency”. Thirdly, the actor-network theory proved the importance of smooth communication between actors in maintaining the system’s resilience from the perspective of “translation”. Finally, this paper investigated the governance process of the social-ecological system of Chang-shan Archipelago from the perspective of actor-network theory, providing a new theoretical perspective for ecological environment governance.

## Figures and Tables

**Table 1:** Basic information of interviewed fishing households.

Basic Information	Attributes	Number of Samples
Family members	2	4
	3	5
	4	21
Means of making a living	Fishing	5
	Mariculture	7
	Fishing and mariculture	18
Annual household income	50,000 to 100,000 yuan	4
	100,000 to 200,000 yuan	21
	More than 200,000 yuan	5
Region	Shicheng island	9
	Dachangshan island	8
	Xiaochangshan island	5
	Zhangzi island	5
	Guanglu island	3

**Table 2:** Outline of the interview.

Introductory questions	How many members are there in your family? Are you natives, or did you move here later?
Flow questions	What do you do for a living? What is the annual household income?
Key questions	Do you have some contacts with other fishermen, government officers, and technical professionals in mariculture and fishing, and do you receive timely help or guidance when you encounter problems? How do you communicate with other fishermen, government officers, and technical professionals? Have mariculture and fishing tools or technologies improved in recent years, and have they greatly affected the fishing harvest? Does the government-organized release have a huge impact on the fishing harvest? Does the government’s fishing moratorium have some effect on restoring the marine environment and fish stocks? Does the government have a specific marine environment protection policy?
Final questions	Can you comprehensively evaluate your feelings about the island’s ecological environment and changes in mariculture and fishing in recent years?

**Table 3:** Open coding of data extracts.

Data Extracts	Coded For
We depend on the sea, as there is more fish and there is more harvest; We mainly rely on fishing and do part-time jobs during the fishing moratorium.	The connections between fishermen and marine creatures
Technical professionals from Dalian Ocean University regularly explained mariculture knowledge to fishermen and solved practical problems encountered in mariculture.	The connections between fishermen and fishing technologies
When fishermen are in danger at sea, they call the rescue number, and the government will organize rescue in time; The government will promote local aquatic products and expand sales channels.	The connections between fishermen and local government
Fishermen go fishing and buy a fishing machine together; Fishermen share weather conditions and mariculture experience.	The connections between fishermen
The government has set up a fishing moratorium, which has made the number and size of marine creatures larger; The government’s environmental protection policy has significantly improved the environment. After the sewage discharge standard has been raised, the seawater has become clear, and the algal density has decreased.	Government behavior
Last year’s storm made three fishermen stranded at sea; the high winds in June this year blew away many of the cages used for sea cucumber culture.	Natural disaster
We started to breed new scallops, which are not easy to die in a rainstorm or extreme weather; the newly developed net-lifter can protect fishing nets to a large extent.	Technological improvement
In the last 2 years, a pilot fishery cooperative has been carried out in the village; Fishery cooperative regularly organizes technical training, technical exchange, and consulting services; When buying fishing machines, sea cucumber, and oyster seedlings, they can bargain collectively with the sellers and can get more favorable prices.	Fishery cooperative
The WeChat group of fishermen regularly shares gale warnings; the WeChat group often shares high-quality and cheap machines, new species and experiences of mariculture; some of the problems that fishermen encounter in mariculture and fishing can also be solved in the WeChat group.	WeChat group of fishermen
The group of villagers will hold regular meetings to explain the new policies implemented by the local government; Fishermen can express their views on the development of the village at the meeting; some conflicts among fishermen can also be solved at the meeting.	Group of villagers

**Table 4:** Presentation of themes and sub-themes.

Themes	Sub-Themes
The connections between fishermen and marine creatures	Heterogeneous networks
The connections between fishermen and fishing technologies	
The connections between fishermen and local government	
The connections between fishermen	
Government behavior	Agency
Natural disaster	
Technological improvement	
Fishery cooperative	Translation
WeChat group of fishermen	
Group of villagers	

## Data Availability

The data for this research were retrieved from the Dalian Statistics Year Book. The data are openly available at http://www.stats.dl.gov.cn/col/col3811/index.html, accessed on 18 November 2022.

## References

[1] Stone-Jovicich S., Goldstein B.E., Brown K., Plummer R., Olsson P. (2018). Expanding the contribution of the social sciences to social-ecological resilience research. Ecol. Soc..

[2] Brand F.S., Jax K. (2007). Focusing the meaning(s) of resilience: Resilience as a descriptive concept and a boundary object. Ecol. Soc..

[3] Lehtonen M. (2004). The environmental-social interface of sustainable development capabilities, social capital, institutions. Ecol. Econ..

[4] Stone-Jovicich S. (2015). Probing the interfaces between the social sciences and social-ecological resilience: Insights from integrative and hybrid perspectives in the social sciences. Ecol. Soc..

[5] Hobman E.V., Walker I. (2015). Stasis and change: Social psychological insights into social-ecological resilience. Ecol. Soc..

[6] Fabinyi M., Evans L., Foale S.J. (2014). Social-ecological systems, social diversity, and power: Insights from anthropology and political ecology. Ecol. Soc..

[7] Boonstra W.J. (2016). Conceptualizing power to study social-ecological interactions. Ecol. Soc..

[8] Janssen M.A., Bodin Ö., Anderies J.M., Elmqvist T., Ernstson H., Mcallister R.R.J., Olsson P., Ryan P. (2006). Toward a network perspective of the study of resilience in social-ecological systems. Ecol. Soc..

[9] Cinner J.E., Barnes M.L. (2019). Social Dimensions of Resilience in Social-Ecological Systems. One Earth.

[10] Bennett N.J., Roth R., Klain S.C., Chan K., Clark D.A., Cullman G., Epstein G., Nelson M.P., Stedman R., Teel T.L. (2017). Mainstreaming the social sciences in conservation. Conserv. Biol..

[11] Holling C.S. (1973). Resilience and Stability of Ecological Systems. Annu. Rev. Ecol. Syst..

[12] Berkes F., Folke C., Colding J. (2000). Linking Social and Ecological Systems: Management Practices and Social Mechanisms for Building Resilience.

[13] Carpenter S.R., Gunderson L.H. (2001). Coping with collapse: Ecological and social dynamics in ecosystem management. Bioscience.

[14] Jozaei J., Chuang W.-C., Allen C.R., Garmestani A. (2022). Social vulnerability, social-ecological resilience and coastal governance. Glob. Sustain..

[15] Biggs R., Schlüter M., Schoon M.L. (2015). Principles for Building Resilience: Sustaining Ecosystem Services in Social-Ecological Systems.

[16] Gonzalez-Quintero C., Avila-Foucat V.S. (2019). Operationalization and Measurement of Social-Ecological Resilience: A Systematic Review. Sustainability.

[17] Janssen M.A., Ostrom E., Tesfatsion L., Judd K.L. (2006). Governing Social-Ecological Systems. Handbook of Computational Economics.

[18] Sundstrom S.M., Allen C.R. (2019). The adaptive cycle: More than a metaphor. Ecol. Complex..

[19] Salvia R., Quaranta G. (2015). Adaptive Cycle as a Tool to Select Resilient Patterns of Rural Development. Sustainability.

[20] Fath B.D., Dean C.A., Katzmair H. (2015). Navigating the adaptive cycle: An approach to managing the resilience of social systems. Ecol. Soc..

[21] Berkes F., Jolly D. (2002). Adapting to climate change: Social-ecological resilience in a Canadian Western Arctic community. Conserv. Ecol..

[22] Rockström J., Steffen W., Noone K., Persson Å., Chapin F.S., Lambin E.F., Lenton T.M., Scheffer M., Folke C., Schellnhuber H.J. (2009). A safe operating space for humanity. Nature.

[23] Adger W.N., Hughes T.P., Folke C., Carpenter S.R., Rockstrom J. (2005). Social-ecological resilience to coastal disasters. Science.

[24] Latour B. (2007). Reassembling the Social: An Introduction to Actor-Network-Theory.

[25] Callon M., Wright J.D. (2001). Actor Network Theory. International Encyclopedia of the Social & Behavioral Sciences.

[26] Murdoch J. (1998). The spaces of actor-network theory. Geoforum.

[27] Callon M. (1999). Actor-network theory—the market test. Sociol. Rev..

[28] Brown S.D. (2002). Michel Serres: Science, translation and the logic of the parasite. Theory Cult. Soc..

[29] Callon M., Knorr W.R., Krohn R., Whitley R.P. (1980). Struggles and negotiations to define what is problematic and what is not. The Social Process of Scientific Investigation.

[30] Walsham G., Lee A.S., Liebenau J., DeGross J.I. (2012). Actor-network theory and IS research: Current status and future prospects. Information Systems and Qualitative Research.

[31] Shen P., Li J.Q. (2021). The Hotspots and Trends of Actor-Network Theory. J. Dialectics Nat..

[32] Smith R.G. (2003). World city actor-networks. Prog. Hum. Geogr..

[33] Murdoch J. (1997). Inhuman/Nonhuman/Human: Actor-Network Theory and the Prospects for a Nondualistic and Symmetrical Perspective on Nature and Society. Environ. Plan. D Soc. Space.

[34] Latour B. (1996). On actor-network theory: A few clarifications. Soz. Welt..

[35] Latour B. (2013). An Inquiry into Modes of Existence.

[36] Blok A., Jensen C.B. (2019). The Anthropocene event in social theory: On ways of problematizing nonhuman materiality differently. Sociol. Rev..

[37] Hartwick E.R. (2000). Towards a geographical politics of consumption. Environ. Plan. A.

[38] Sayes E. (2017). Marx and the critique of Actor-Network Theory: Mediation, translation, and explanation. Distinktion J. Soc. Theory.

[39] Billson J.M. (1989). Focus groups: A practical guide for applied research. Clin. Sociol. Rev..

[40] Braun V., Clarke V. (2006). Using thematic analysis in psychology. Qual. Res. Psychol..

[41] Clarke V., Braun V. (2013). Teaching thematic analysis: Overcoming challenges and developing strategies for effective learning. Psychol..

[42] Karlsen M.-M.W., Gabrielsen A.K., Falch A.L., Stubberud D.-G. (2017). Intensive care nursing students’ perceptions of simulation for learning confirming communication skills: A descriptive qualitative study. Intensive Crit. Care Nurs..

[43] Anderies J.M., Janssen M.A., Ostrom E.A. (2004). Framework to Analyze the Robustness of Social-ecological Systems from an Institutional Perspective. Ecol. Soc..

[44] Ostrom E. (2007). A diagnostic approach for going beyond panaceas. Proc. Natl. Acad. Sci. USA.

[45] Ostrom E. (2009). A General Framework for Analyzing Sustainability of Social-Ecological Systems. Science.

[46] Fidel M., Kliskey A., Alessa L., Sutton O.P. (2014). Walrus harvest locations reflect adaptation: A contribution from a community-based observation network in the Bering Sea. Polar Geogr..

[47] Cressman D. A Brief Overview of Actor-Network Theory: Punctualization, Heterogeneous Engineering & Translation. https://summit.sfu.ca/item/13593.

[48] Simons M. (2017). The parliament of things and the Anthropocene: How to listen to ‘quasi-objects’. Techné Res. Philos. Technol..

[49] Walker B., Gunderson L., Kinzig A., Folke C., Carpenter S., Schultz L. (2006). A handful of heuristics and some propositions for understanding resilience in social-ecological systems. Ecol. Soc..

[50] Holling C.S. (2001). Understanding the Complexity of Economic, Ecological, and Social Systems. Ecosystems.

[51] Holling C.S. (1986). The resilience of terrestrial ecosystems: Local surprise and global change. Sustain. Dev. Biosph..

[52] Chaffin B.C., Gunderson L.H., Allen C.R., Garmestani A. (2022). Panarchy and the Governance of Social-Ecological Systems. Applied Panarchy: Applications and Diffusion Across Disciplines.

[53] Holling C.S., Gunderson L.H. (2002). Panarchy: Understanding Transformations in Human and Natural Systems.

[54] Carpenter S., Walker B., Anderies J.M., Abel N. (2001). From metaphor to measurement: Resilience of what to what?. Ecosystems.

[55] De Kraker J. (2017). Social learning for resilience in social-ecological systems. Curr. Opin. Environ. Sustain..

[56] Rigolot C., De Voil P., Douxchamps S., Prestwidge D., Van Wijk M., Thornton P.K., Rodriguez D., Henderson B., Medina D., Herrero M. (2017). Interactions between intervention packages, climatic risk, climate change and food security in mixed crop-livestock systems in Burkina Faso. Agric. Syst..

[57] Haque C.E., Berkes F., Fernández-Llamazares Á., Ross H., Iii F.S.C., Doberstein B., Reed M.G., Agrawal N., Nayak P.K., Etkin D. (2021). Social learning for enhancing social-ecological resilience to disaster-shocks: A policy Delphi approach. Disaster Prev. Manag. Int. J..

[58] Mukhovi S.M., Jacobi J., Llanque A., Rist S., Delgado F., Kiteme B., Speranza C.I. (2020). Social self-organization and social-ecological resilience in food systems: Lessons from smallholder agriculture in Kenya and indigenous Guaraní communities in Bolivia. Food Stud..

[59] Moyson S., Scholten P., Weible C.M. (2017). Policy learning and policy change: Theorizing their relations from different perspectives. Policy Soc..

[60] De Vries D.H. (2017). Temporal vulnerability and the post-disaster ‘Window of Opportunity to Woo:’a case study of an African-American floodplain neighborhood after Hurricane Floyd in North Carolina. Hum. Ecol..

[61] Calvet-Mir L., Riu-Bosoms C., González-Puente M., Ruiz-Mallén I., Reyes-García V., Molina J.L. (2016). The Transmission of Home Garden Knowledge: Safeguarding Biocultural Diversity and Enhancing Social-Ecological Resilience. Soc. Nat. Resour..

[62] Peterson G., Allen C.R., Holling C.S. (1998). Ecological resilience, biodiversity, and scale. Ecosystems.

[63] Di Falco S., Chavas J.-P. (2008). Rainfall Shocks, Resilience, and the Effects of Crop Biodiversity on Agroecosystem Productivity. Land Econ..

[64] Ruiz-Ballesteros E., Ramos-Ballesteros P. (2019). Social-Ecological Resilience as Practice: A Household Perspective from Agua Blanca (Ecuador). Sustainability.

[65] Booher D.E., Innes J.E. (2010). Governance for Resilience: CALFED as a Complex Adaptive Network for Resource Management. Ecol. Soc..

[66] Ford J.D., King N., Galappaththi E.K., Pearce T., McDowell G., Harper S.L. (2020). The Resilience of Indigenous Peoples to Environmental Change. One Earth.

[67] Brown K., Westaway E. (2011). Agency, Capacity, and Resilience to Environmental Change: Lessons from Human Development, Well-Being, and Disasters. Annu. Rev. Environ. Resour..

[68] Chaffin B.C., Gunderson L.H. (2016). Emergence, institutionalization and renewal: Rhythms of adaptive governance in complex social-ecological systems. J. Environ. Manag..

[69] Sayes E. (2013). Actor–Network Theory and methodology: Just what does it mean to say that nonhumans have agency?. Soc. Stud. Sci..

[70] Nabavi E., Daniell K.A. (2017). Rediscovering social–ecological systems: Taking inspiration from actor-networks. Sustain. Sci..

[71] Ruikar S., Chang P.C. (2012). Achieving Network Stability through Convergence--Case Study of an E-Government Project Using Actor Network Theory. Proceedings of the 2012 45th Hawaii International Conference on System Sciences.

[72] Law J., Turner B.S. (2009). Actor network theory and material semiotics. The New Blackwell Companion to Social Theory.

[73] Young O.R. (2002). The Institutional Dimensions of Environmental Change: Fit, Interplay, and Scale.

[74] Chaffin B.C., Gosnell H., Cosens B.A. (2014). A decade of adaptive governance scholarship: Synthesis and future directions. Ecol. Soc..

[75] Pahl-Wostl C., Knieper C. (2014). The capacity of water governance to deal with the climate change adaptation challenge: Using fuzzy set Qualitative Comparative Analysis to distinguish between polycentric, fragmented and centralized regimes. Glob. Environ. Change-Hum. Policy Dimens..

[76] Lemos M.C., Agrawal A. (2006). Environmental governance. Annu. Rev. Environ. Resour..

